# A Unique Case for Spinal Cord Stimulation: Successful Treatment of Small Fiber Neuropathy Pain Using Multiple Spinal Cord Stimulators

**DOI:** 10.1155/2017/6969285

**Published:** 2017-07-16

**Authors:** Maxim Eckmann, Alexander Papanastassiou, Mark Awad

**Affiliations:** University of Texas Health Sciences Campus in San Antonio (UTHSCSA), San Antonio, TX, USA

## Abstract

Spinal cord stimulators have commonly been used to treat multiple pain conditions. This case report represents a unique case of using multiple spinal cord stimulators for widespread small fiber neuropathy pain. This case report concerns patient JJ who first presented with generalized neuropathic pain. His pain was an intermittent burning, stinging quality that originally focused in both of his feet and progressed to include his legs and arms and eventually involved his entire body. The pain would last moments to hours at least daily. He reported a poor quality of life. He was diagnosed with small fiber neuropathy with anhydrosis, suggestive of idiopathic erythromelalgia. He had a spinal cord stimulator trial involving both cervical and lower thoracic percutaneous leads. After two spinal cord stimulators were implanted, the patient began to report an improvement in pain. The patient continues to report excellent pain relief. The patient uses the stimulator intermittently as needed, in an abortive fashion for pain flares. The patient is very pleased and has increased his activity. He now attends graduate school full time. This case report hopes to illustrate a unique use of multiple spinal cord stimulators in treating widespread neuropathic pain caused by small fiber neuropathy.

## 1. Introduction

Neuromodulation has been classically defined by the International Neuromodulation Society as “the alteration of nerve activity through targeted delivery of a stimulus, such as electrical stimulation or chemical agents, to specific neurological sites in the body” [1] and spinal cord stimulation is a type of neuromodulation where epidural leads are placed in the body and attached to an implanted pulse generator to create a current that helps modify different types of neural signals. In our context, the desire is to decrease pain signaling.

Spinal cord stimulators (SCS) have been commonly used to treat multiple pain conditions including complex regional pain syndrome (CRPS), failed laminectomy/failed back surgery syndrome (FBSS), and severe low back pain refractory to other treatments [2]. Now as the use of spinal cord stimulators has increased so have the potential indications. Recently, SCS have been used to effectively treat diabetic peripheral neuropathy [3]. SCS has increasingly been used in various neuropathic pain syndromes when standard treatments had already failed [4]_._ SCS is already being used for small fiber neuropathy. In 2015, a retrospective study by Hayek et al. found that out of the 345 patients who underwent SCS trial, 18 patients had a SCS implanted for small fiber neuropathic pain [5]. Also, in 2017, Maino et al. described a case of a patient with small fiber neuropathy whose pain was treated successfully with spinal cord stimulation but that was using dorsal root ganglion stimulation and treating pain in only one limb (left foot) [6]. While these studies help to establish SCS as a reliable treatment for small fiber neuropathic pain, our case report hopes to illustrate a unique use of two SCS implants and four leads to treat whole body pain in a patient with small fiber neuropathy.

Small fiber neuropathy is a sensory neuropathy manifested usually by painful paresthesias, along with findings of abnormal small fiber nerve function seen on neurological examination, specialized electrodiagnostic testing, and/or pathological studies. Common etiologies include diabetes, B12 deficiency, metabolic disorders, and autoimmune disorders. It is commonly treated with medications such as antidepressants (TCAs and SSRIs), anticonvulsants, opioids, and topical capsaicin [7]. There are also case reports and studies of spinal cord stimulation being used to successfully treat small fiber neuropathic pain; few however have included dual implants with one set of leads and battery to treat the upper extremity pain and another set of leads and battery for the lower extremity.

## 2. Methods

This case report concerns patient JJ who first presented to a neurologist for generalized neuropathic pain in 2013. On August 7, 2013, patient JJ was a twenty-year-old male and visited neurology for the first time that day. He had already suffered numbness and tingling in his feet with sharp stinging pain that had progressed into his legs, hands, arms, trunk, and face. The pain started about a year earlier. His triggers included heat and physical activity and lifting things for a long period of time or high intensity short bursts of activity would cause the pain. Pain episodes would also happen while he was sleeping and wake him up. At that point, he had already tried gabapentin, pregabalin, and escitalopram and he was on buprenorphine and fentanyl patches with still no relief. An MRI of his brain and cervical spine were normal.

The neurologist stated that the history was suggestive of small fiber neuropathy but that other differentials had to be ruled out. A NCS/EMG, skin biopsy, and multiple labs were ordered. The NCS showed no evidence of large fiber neuropathy and the other labs helped to rule out other differential causes. Quantitative sudomotor axon reflex testing (QSART) showed reduced sweat volumes at all sites with a non-length dependent reduction providing evidence of non-length dependent painful small fiber neuropathy. The neurologist determined that JJ had small fiber neuropathy with anhydrosis, suggestive of idiopathic erythromelalgia. A trial of prednisolone was attempted but patient had no relief of pain. By this time, patient JJ stopped the buprenorphine and fentanyl patches and had no relief with other medications including carbamazepine, oxcarbazepine, or duloxetine. JJ began to report a poor quality of life. He felt unable to go to any uncontrolled environments due to the severe episodes of sharp debilitating pain. He, along with his family, was becoming desperate for other pain relief options. His neurologist referred the patient to the UTHSCSA pain management consultants for more options.

On January 27, 2014, JJ presented to our pain clinic for the first time. At that point, we considered JJ to have diffuse episodic neuropathic pain that occurred at least daily and had an unclear etiology. We discussed the various options with the patient including infusion therapy, acupuncture, cognitive behavioral therapy, and spinal cord stimulation. After that initial visit, the patient and family elected to try other pharmacologic options, including orphenadrine, dextromethorphan, and mexiletine. The patient also received two ketamine infusions: 150 mg over one hour on 04/17/2014 and 360 mg over one hour on 09/04/2014. During this time, the patient was still receiving work-up including being referred for whole exome sequencing but no abnormality was found in the relevant genes. JJ wanted to try a lidocaine infusion before SCS so he received a lidocaine infusion on 11/12/15. Unfortunately, the lidocaine infusions like all the previous therapies did not offer him much pain relief. On November 2015, JJ began to seriously consider SCS therapy and eventually agreed to a SCS trial. Due to his pain pattern with bilateral upper and lower extremity pain, the decision was made to try both cervical and thoracic leads.

On February 9, 2016, our clinic performed a SCS trial with one lead tip ending at the top of the T8 vertebral body level and a second lead tip at the top of the C3 vertebral body level. JJ had a great response with the trial and wanted to move on to implantation.

## 3. Results

**Figure 1:**
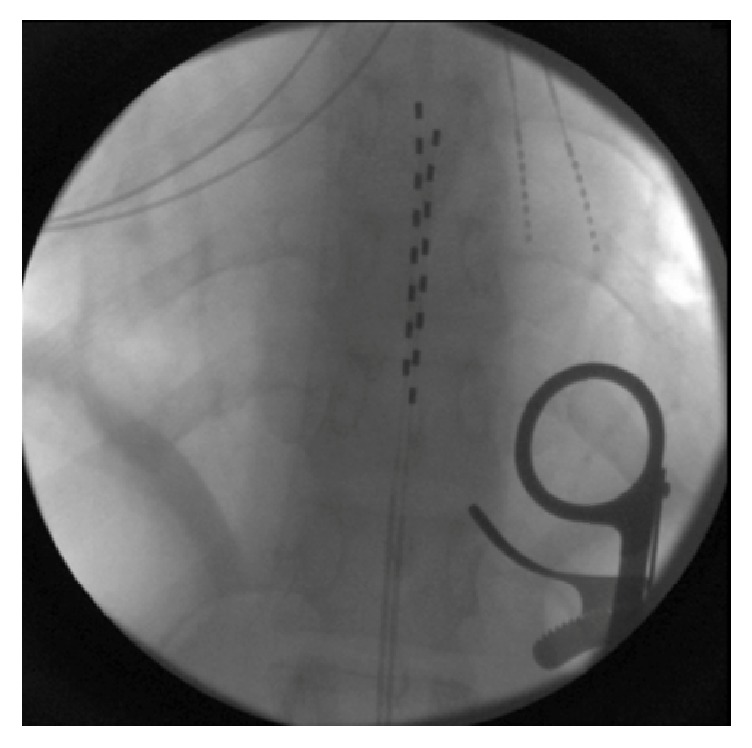
Two 8-contact epidural leads lying in their final position in the mid-thoracic spine.

**Figure 2:**
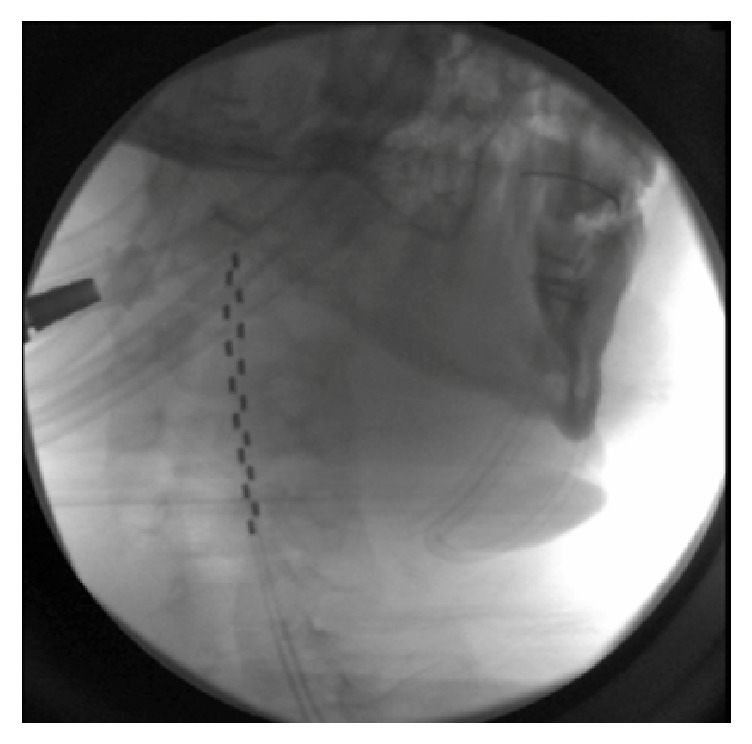
Two 8-contact epidural leads from the spinal cord stimulator lying in their final position in the cervical spine.

On March 22, 2016, JJ had two SCS generators and two cervical and two thoracic leads implanted in conjunction with neurosurgeon Dr. Papanastassiou (Figures 1 and 2).

After the two spinal cord stimulators and generators were implanted, the patient began to report an improvement in pain. This stimulation was tonic and paresthesia based. The patient continued to report excellent pain relief six months after implantation. Within six months, patient had 100% abolition of pain in his extremities and lower body half while using stimulation. This represented about 90% of his overall pain. The stimulation covered his arms, his waist down to his legs, and most of his trunk with one small gap. The patient uses the stimulator intermittently as needed, in an abortive fashion for pain flares. JJ had had no complaints of autonomic or erythromelalgia symptoms.

The patient was extremely pleased and increased his activity. He is no longer taking any analgesic or antiepileptic medications. He is able to ride his scooter and wear a backpack and now attends graduate school full time.

## 4. Discussion

Plenty of discussion and research has been done on treatment of FBSS, CRPS, and axial low back pain through neuromodulation but very little literature exists about the possible role of neuromodulation and specifically SCS in the treatment of small fiber neuropathy.

Patient JJ had a long and complicated medical course and underwent over three years of medical testing, including numerous imaging studies as well as invasive tests like skin biopsies. Earlier neuromodulation intervention could have saved him years of suffering. It is debatable whether the implantation of the two batteries is cost-effective. However, it is clear that neuromodulation has done more for the patient's refractory pain than any other treatment while also avoiding the risks associated with medications and side effects. In addition, JJ's use of spinal cord stimulation in an abortive manner is a unique use of the therapy. Typically, patients have their SCS on throughout the day, changing modes as needed, but JJ seemed to only need SCS before attacks to prevent them or during attacks to stop them.

This case report indicates a small sample of what is possible with neuromodulation and the emerging role neuromodulation can play in addressing atypical and neuropathic pain conditions. As previously stated, SCS is already being used for diabetic and small fiber neuropathy pain. In 2015, a retrospective study by Hayek et al. found that, out of the 345 patients who underwent SCS trial, 18 patients had a SCS implanted for small fiber neuropathic pain [5]. Also, in 2017, Maino et al. described a case report of a patient with small fiber neuropathy whose pain was treated successfully with spinal cord stimulation but that was using dorsal root ganglion stimulation and treating pain in only one limb (left foot) [6]. While the Hayek et al. study and Maino et al. case report help to establish SCS as a known treatment for small fiber neuropathic pain, our case report hopes to illustrate a unique use of multiple spinal cord stimulators in treating widespread neuropathic pain caused by small fiber neuropathy [5, 6].
